# Sunitinib Combined with Angiotensin-2 Type-1 Receptor Antagonists Induces More Necrosis: A Murine Xenograft Model of Renal Cell Carcinoma

**DOI:** 10.1155/2014/901371

**Published:** 2014-05-22

**Authors:** Grégory Verhoest, Thibault Dolley-Hitze, Florence Jouan, Marc-Antoine Belaud-Rotureau, Emmanuel Oger, Audrey Lavenu, Karim Bensalah, Yannick Arlot-Bonnemains, Nicolas Collet, Nathalie Rioux-Leclercq, Cécile Vigneau

**Affiliations:** ^1^CNRS/UMR 6290/Biosit, Rennes 1 University, 35043 Rennes Cedex, France; ^2^CHU Rennes, Department of Urology, 35033 Rennes, France; ^3^Rennes University Hospital, 2 rue Henri Le Guilloux, 35033 Rennes Cedex, France; ^4^CHU Rennes, Department of Nephrology, 35033 Rennes, France; ^5^CHU Rennes, Department of Cytogenetics, 35033 Rennes, France; ^6^Rennes 1 University, 35043 Rennes, France; ^7^CHU Rennes, Department of Clinical Pharmacology, 35033 Rennes, France; ^8^INSERM/Pharmacoepidemiology Team/CIC0203/Biosit, Rennes 1 University, 35043 Rennes, France; ^9^CHU Rennes, Department of Biochemistry, 35033 Rennes, France; ^10^CHU Rennes, Department of Pathology, 35033 Rennes, France

## Abstract

*Background*. Angiotensin-2 type-1 receptor antagonists not are only antihypertensive drugs but also can inhibit VEGF production. We hypothesised that adding telmisartan to sunitinib could potentiate the antiangiogenic effects. *Material and Methods*. 786-O cell lines were injected in nude mice. After tumor development, mice were divided into 4 groups: the first was the control group (DMSO), the second group was treated with sunitinib alone, the third group was treated with telmisartan alone, and the fourth group was treated with the combination. Drugs were orally administered every day for four weeks. Animals were sacrificed after treatment. Blood and tumor tissues were collected for analysis by immunohistochemistry, Western Blot, and ELISA methods. *Results*. All animals developed a ccRCC and ten in each group were treated. Using a kinetic model, tumors tended to grow slower in the combination group compared to others (*P* = 0.06). Compared to sunitinib alone, the addition of telmisartan significantly increased tissue necrosis (*P* = 0.038). Central microvascular density decreased (*P* = 0.0038) as well as circulating VEGF (*P* = 0.003). There was no significant variation in proliferation or apoptosis markers. *Conclusion*. The combination of sunitinib and telmisartan revealed an enhancement of the blockage of the VEGF pathway on renal tumor resulting in a decrease in neoangiogenesis and an increase in necrosis.

## 1. Introduction

Clear cell renal cell carcinoma (ccRCC) represents almost 70% of renal cancers and 3% of malignant tumours in adults. Distant metastases are present in 20% of the cases, and 30% of the patients will develop metastases during their natural history [1]. One of the bad prognostic factors for this tumour is a high vascular endothelial growth factor (VEGF) concentration, which stimulates neoangiogenesis [2]. During the last years, new antiangiogenic treatments targeting tyrosine kinase receptors like VEGF receptors have improved patients' survival [3, 4]. Nevertheless, many patients are likely to develop cardiac (hypertension or cardiac failure) or renal side effects (proteinuria and thrombotic microangiopathy), compelling them to reduce or stop these treatments. However, hypertension development could predict a good response to antiangiogenic therapies [5, 6].

Angiotensin II is the active peptide of the Renin-Angiotensin System (RAS). It is a major regulator of blood pressure and cardiovascular homeostasis. Its biological effects are mainly mediated through two types of receptors: AT1-R and AT2-R, and most physiological effects have been attributed to AT1-R stimulation. Angiotensin II has been shown to play a role in different pathological situations involved in tissue remodelling like cardiac hypertrophy, development, or wound healing and also in cancers [7].

In addition, several experimental models recently suggested that angiotensin II could be involved in cancer development and progression [8, 9] and that RAS blockage by AT1-R antagonists or Angiotensin converting enzyme inhibitors (ACE-Is) could be useful in cancer therapy [10]. We therefore hypothesized that such combination could be tested in ccRCC so that AT1-R antagonists could potentiate the effects of tyrosine kinase inhibitors. We also recently demonstrated that AT1-R was overexpressed in ccRCC with high Fuhrman grade and that this overexpression was correlated to patients' survival [11]. These data lead us to suppose that AT1-R blockers could have antiangiogenic and/or antiproliferative and/or proapoptotic properties in ccRCC treatment.

The objective of this study was to evaluate the impact of one AT1-R blocker, telmisartan, alone or in combination with one tyrosine kinase inhibitor (TKI), sunitinib, on ccRCC from a macroscopic to a biological perspective.

## 2. Material and Methods

### 2.1. Materials

Sunitinib was generously supplied by Pfizer (SU-11248 AKA PF-2783926-41, Pfizer, USA). Telmisartan and DMSO were from Sigma-Aldrich, USA.

### 2.2. Murine Xenograft Model of ccRCC and Treatments

The scientific project got all permissions from an ethical committee, which had a full description of the project, to ensure minimum pain among the animals (Rennes University: R-2010-CV-01 and R-2011-CV-01). All mice were maintained according to the guide for the care and use of laboratory animals.

Forty female nude athymic BALB/c mice (Janvier Laboratory, France) between 6 and 8 weeks were injected with 10^7^ cells 786-O (Human commercial ccRCC cells lines, ATCC, USA) subcutaneously in the left flank as previously described [12].

Mice were weighed and blood pressure was measured by a plethysmographic and noninvasive device (CODA STANDARD MONITOR, Emka Technologies, USA) once a week and also during all of the duration of the experiment. The growth of tumor xenografts was monitored once a week by caliper measurement of length (*a*), width (*b*), and thickness (*c*). Tumor volumes were calculated using the formula (*a* ×* b* ×* c*) × 0.5236.

Five weeks after tumor cell injection, mice were divided into 4 groups of 10 mice according to the following plan: the first group comprising 10 untreated mice used as a control group (DMSO, injection), the second group was composed of 10 mice treated with sunitinib alone (40 mg/kg/d), the third group included 10 mice treated with ARA-2 alone (telmisartan 5 mg/kg/d), and the fourth consisted of 10 mice treated with the combination of sunitinib (40 mg/kg/d) and ARA-2 (telmisartan 5 mg/kg/d). Sunitinib or telmisartan powders were dissolved in pure DMSO, then diluted in a 1% water solution, and sonicated. Previous studies showed that the bioavailability and the metabolisation of sunitinib were similar for mouse and human allowing us to treat mice with 40 mg/kg/d [13]. Telmisartan was delivered at 5 mg/kg/d according to previous studies [14]. At the end of the 4-week treatments, mice were anesthetized 24 hours after the last drug administration with a solution of ketamine (Imalgene 1000, Merial, France) and xylazine 2% (Rampun 2%, Bayer, Germany) administered subcutaneously in thigh. Blood was immediately removed by intracardiac puncture. Serum was frozen at −80°C for further analysis after blood clotting and 8000 g centrifugation. Tumors were harvested and kept for analysis. Tumor tissues were divided into two equal parts, frozen in liquid nitrogen or fixed in a 10% formaldehyde solution, and embedded in paraffin.

### 2.3. Immunofluorescence (IF)

Blocking has been performed with immunofluorescence buffer (PBS, 3% BSA, 0.2% Triton 100x) for 1 h followed by 15 min incubation with a second immunofluorescence buffer (PBS, 1% BSA, 0.06% Triton 100x). Primary antibody against AT1R (1 : 100, Rabbit, (N-10): sc-1173, Santa-Cruz Biotechnology, USA) has been incubated for 2 h at room temperature in a humidified chamber. After washing, cells have been incubated with Alexa-Fluor 546 goat anti-Rabbit Ig G (H + L), (1 : 5000, Molecular Probes, USA) for 2 hours. The nuclei were counterstained with DAPI/Antifade (Q-Biogene, MP Biomedicals, USA). Samples were analyzed with fluorescence microscope system (LEICA DMRXA, Germany).

### 2.4. Histology and Immunochemistry (IHC)

Histological examination was performed on 5 *μ*m formalin-fixed paraffin sections stained with hematoxylin and eosin safran. All specimens have been examined blindly by a single pathologist (NRL).

The IHC experiments were realized on 5 *μ*m tumour sections which were incubated at room temperature for 1 h with primary antibodies against KI67 (1 : 100, monoclonal rabbit antibody, clone SP6, Interchim, France), CD31 (1 : 50, rat monoclonal antibody, clone SZ 31, Dianova, Germany), and VEGF-A (1 : 100, rabbit polyclonal antibody, clone Ab-4, Calbiochem, USA). Immunostaining was performed using BenchMark XT-Ventana Medical Systems with kit DABMAP (using streptavidin/biotin system) for CD31 or kit OMNIMAP (system “biotin-free” using multimer technology) for KI67 and VEGF with antigen retrieval for all (citrate buffer pH 6.0; Tris/Borate/EDTA pH 8.0). In order to detect apoptotic cells, tumors were stained with antiactivated caspase-3 primary antibody (1 : 500, monoclonal mouse antibody, clone 3G2, Cell Signaling Technology, USA). Tissues have been blocked for 15 min and then incubated with primary antibody overnight at 4°C, and the incubation with the secondary antibody conjugated to horseradish peroxidase (Envision + Dual Link System-HRP, Dako, Denmark). Revelation was performed with the diaminobenzidine chromogen (Dako, Denmark). Negative controls were performed by omitting the primary antibody. Immunoreactivity of VEGF-A, KI67, and activated caspase-3 was expressed as the percentage of positive cells by scoring 500 cells. MicroVascular Density (MVD), expressed as the number of vessel sections per mm², was determined by analyses of 10 representative fields accounting for 2 mm². To perform the analysis of tumor necrosis, glass slides were converted to digital slides with the scanner Nanozoomer 2.0-RS Hamamatsu. We used the NDP viewer Hamamatsu to determine the percentage of necrosis by measurement of tumor area and necrosis, respectively. The way is to draw a freehand region around the tissue of interest to obtain an automatic area measurement.

### 2.5. Measurement of VEGF by ELISA

VEGF concentrations of the murine serum were determined by ELISA method in accordance with supplier's instructions (Quantikine kit, RnD, USA).

### 2.6. Western Blot Analysis

Protein extracts were prepared from frozen tumor tissues. Thin cuts of tissues were done before homogenisation in mRIPA buffer (50 mM Tris-HCl, pH 7.4, 1% NP-40, 0.5% sodium deoxycholate, 150 mM sodium chloride, 1 mM EDTA, 1 mM sodium fluoride, 1 mM AEBSF, 10 *μ*g/mL aprotinin, 10 *μ*g/mL leupeptin, and 1 mM sodium orthovanadate). Extracts were centrifuged at 10,000 rpm for 10 min and centrifuged during 30 min at 4°C. Fifty *μ*g of each extract was electrophoresed on a 15% polyacrylamide gel and transferred onto nitrocellulose membranes. The membranes washed with TBST (50 mM Tris-HCl, pH 7.5, 150 mM NaCl, 0.05% Tween-20) have been saturated with 5% low fat milk in TBST for 2 h at room temperature and then incubated with primary antibodies in 2.5% low fat milk in TBST at 4°C overnight, followed by an incubation with horseradish peroxidase conjugated anti-rabbit or anti-mouse IgG antibodies (1 : 50 000 in TBST-BSA2.5% for 1 h at RT). All the protein extracts were frozen at −80°C before blotting.

Antibodies against p44/42 MAP kinase (Erk1/2) (1 : 2000, polyclonal rabbit antibody, Cell Signaling, USA), phospho-p44/42 MAPK (Erk1/2) (Thr202/Tyr204) (1 : 1000, monoclonal mouse antibody, E10 clone, Cell Signaling, USA), AKT (1 : 500, polyclonal rabbit antibody, 9272, Cell Signaling, USA), phospho-AKT (Ser 473) (1 : 1000, polyclonal rabbit antibody, Cell Signalling), phospho-VEGFR2/KDR/flk-1 (Y1214) (1 : 1000, polyclonal rabbit antibody, RnD Systems, USA), and PARP (1 : 500, monoclonal rabbit antibody, 46D11 clone, Cell Signaling, USA) were incubated overnight at 4°C, and then horseradish peroxidase-conjugated secondary antibodies (1 : 10000, Jackson Immuno-Research laboratories, Baltimore, MD) that specifically bind to the primary antibody were used. The blots were then disclosed with a chemiluminescent detection system (SuperSignal West Dura Extended Duration Substrate, Pierce Biotechnology, USA) which can be visualised on X-ray film (Tabletop processor, CURIX 60, AGFA HealthCare, Belgium).

### 2.7. MTT Assay for Cell Viability

The effect of the sunitinib, telmisartan, or combined association on cell proliferation was assayed in sterile 96-well microtiter tissue plates (Becton Dickinson, Oxnard, USA). The 786-O cells were seeded at 2.5 × 10^4^ cells/mL of medium (100 *μ*L per well). The different compounds (10 *μ*L per well) at the appropriate concentration (10 nM–1 mM) were added after 24 hours of cell culture. Incubations were performed at 37°C during 72 h. After exposure to the compounds, cell growth was determined by measuring the formazan formation from 3-(4,5-dimethylthiazol-2yl)-2,5-diphenyltetrazolium (MTT). Multiskan MCC/340 microplate reader (Labsystems, Israel) was used for absorbance measurements (570 nm). Viability was determined by calculation of the ratio of optical density at *J*3/*J*0.

### 2.8. Statistical Analysis

Data are expressed as median. Comparison of continuous variables used nonparametric Kruskal-Wallis or Wilcoxon tests. For tumor growth, all individual data were analyzed simultaneously using nonlinear mixed effect models, which allow sharing information across subjects. Correlation between continuous variables was assessed by Spearman correlation. A probability (*P*) value of less than 0.05 was considered statistically significant for overall intergroup comparisons and two-by-two comparisons. Analyses were performed with SAS software version 9.2 (SAS Institute, USA).

## 3. Results

### 3.1. Cell Lines Express Angiotensin Type-1 Receptor

**Figure 1:**
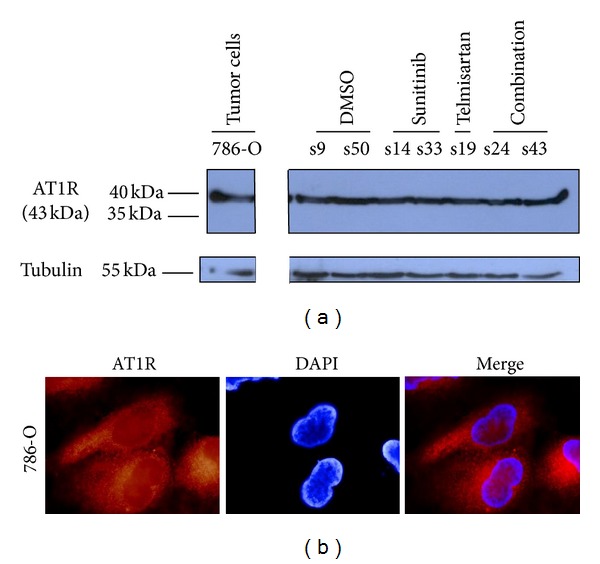
(a) Western Blot analysis of AT1R expression in 786-O cells and in different mice treated by either DMSO, sunitinib, telmisartan, or combined drugs. (b) Immunostaining of AT1R (red) and DAPI staining (blue) in 786-O cells. AT1-R stains for the membranes of tumour and endothelial cells (×1000).

The expression of AT1R was revealed by Western Blot on 786-O cells and tumor tissues treated or not with sunitinib alone, telmisartan alone, or combination (Figure 1(a)). The presence of the receptor AT1-R was visualised onto the cell line 786-O cells by immunofluorescence (Figure 1(b)).

### 3.2. Mice and Tumor Development

Ten mice were treated in each group. Two animals died in the control group before the beginning of treatment. One mouse that belongs to the sunitinib group developed severe side effects as oedema, hypertension, and hepatomegaly and died 10 days after the beginning of treatment. Nevertheless, its tumor was harvested and analyzed. In addition, one mouse corresponding to the group treated with telmisartan did not develop any tumour. Consequently, 3 tumours were missing for the analysis.

During the follow-up, mice treated with sunitinib alone developed hypertension. Medium arterial pressure in the sunitinib group, 100.7 ± 21.8 mmHg, was significantly different from blood pressure in other groups, 85.3 ± 18.0 mmHg in control group, 77.0 ± 18.2 mmHg in the telmisartan group, and 70.8 ± 1.9 mmHg in the association group (*P* = 0.0034, Kruskal-Wallis test). These results confirm that drugs orally administered were well assimilated by animals leading to expected systemic effects on blood pressure.

**Figure 2:**
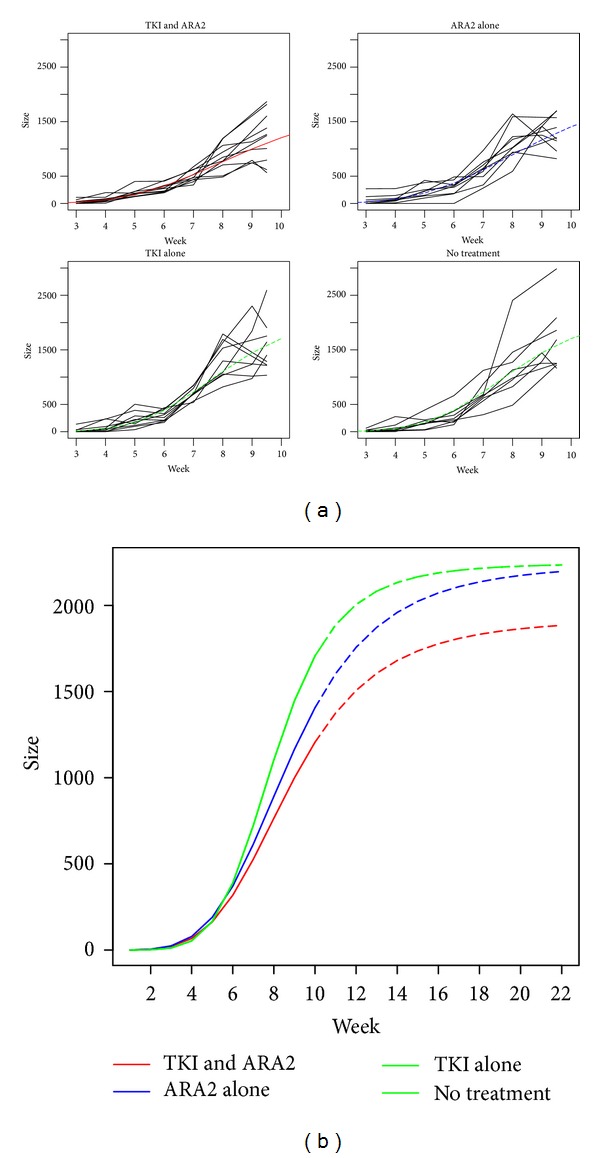
(a) Kinetics of tumor growth separated in four groups with or without telmisartan (ARA2) and sunitinib (TKI). (b) Kinetic curves with prolongation until 22 weeks of the four groups with or without telmisartan (ARA2) and sunitinib (TKI).

Using a statistical kinetic model, tumors tended to grow slower in the combination group compared to other groups (*P* = 0.06) (Figures 2(a) and 2(b)—see Supplementary Data available online at http://dx.doi.org/10.1155/2014/901371).

### 3.3. Combination of Telmisartan with Sunitinib Increases Tumour Necrosis

**Figure 3:**
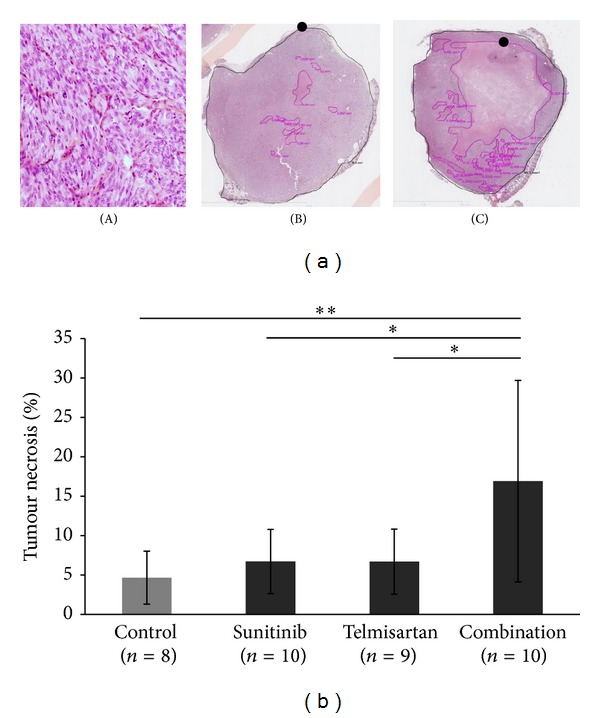
Combination significantly increases tumor necrosis but not sunitinib alone. (a) Histological analysis of tumors by HES staining (×200) reveals Fuhrman 4 ccRCC (A). Tumor necrosis (•) evaluated by HES staining (×20) in tumor from control group (B) and tumor from combination of sunitinib and telmisartan (C). (b) Quantification of necrosis in tumors from different groups. Mean ± SEM, *P < 0.05, **P < 0.02.

The histological analysis of tumors induced in mice revealed in all cases the development of a Fuhrman 4 ccRCC (Figure 3(a)(A)). HES staining also revealed the presence of necrosis area which was significantly more extensive when mice were treated with the combination of telmisartan and sunitinib (*P* = 0.038, Figures 3(a)(B) and 3(a)(C)). Indeed, necrosis in the combination group (16.9% ± 12.8%) was significantly more important compared to the control group (4.7% ± 3.4%—*P* = 0.0185), the sunitinib group (6.7% ± 4.1%—*P* = 0.0376), or the telmisartan group (6.7% ± 4.1%—*P* = 0.0373). As a consequence, the quantity of viable tumour decreased in the combination group compared to the others (*P* < 0.0001).

### 3.4. Combination Inhibits Neovascularisation

**Figure 4:**
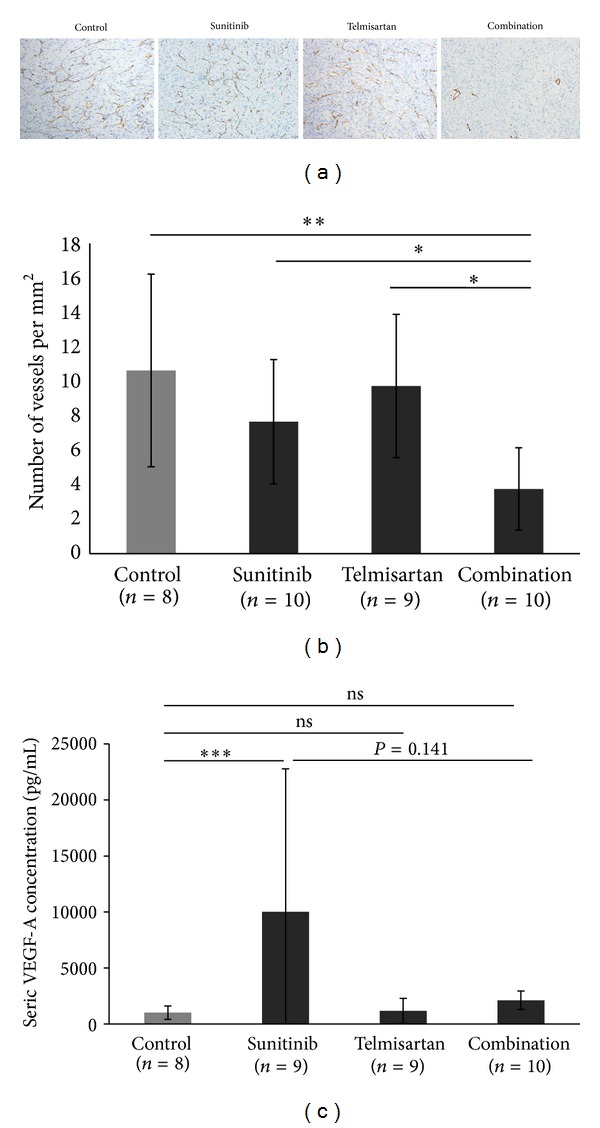
Sunitinib in combination with telmisartan significantly decreases central MVD and tends to decrease seric VEGF-A concentration. (a) Evaluation of central microvascular density by CD31 staining of tumors in a control tumor or after treatment with sunitinib alone, telmisartan alone, and combination (×100). (b) Quantification of central microvascular density as number of vessels per mm^2^. (c) Quantification of seric VEGF-A concentration by ELISA method. Mean ± SEM, *P < 0.05, **P < 0.02, and ***P < 0.001.

As tumour necrosis increased in mice treated with the combination of drugs, we investigated tumour vascularisation. Microvascular density (MVD) was determined by CD31 staining for each tumour (Figure 4(a)). Drugs combined globally decreased MVD in the centre of tumors (MVD = 3.8 vessels/mm² ± 2.4—*P* = 0.0038) as well as compared to control group (10.6 ± 5.6 vessels/mm²—*P* = 0.0029), sunitinib group (7.7 ± 3.6 vessels/mm²—*P* = 0.0171), and telmisartan group (9.7 ± 4.2 vessels/mm²—*P* = 0.0036) (Figure 4(b)). It was also observed that tumors with low MVD had more extensive necrosis (Spearman correlation coefficient is 0.45—*P* = 0.005). In contrast to central vasculature, peripheral vasculature was not affected by drug regimen administered.

### 3.5. Combination Decreases VEGF-A Concentration in Mice Serum

As the drug administration affected tumor vascularisation, we wondered whether the concentration of circulating VEGF-A was altered. We evaluated the circulating VEGF-A concentration by ELISA. The amount of VEGF-A significantly increased in the sunitinib group (10022 pg/mL ± 12741 pg/mL) compared to the control group (1010 pg/mL ± 606 pg/mL—*P* = 0.0045), whereas there was no significant difference with the telmisartan group (1172 pg/mL ± 1122 pg/mL—*P* = 1.00). When telmisartan was used in combination, the upraise of seric VEGF-A concentration induced by sunitinib was lower but not statistically significant (2117 pg/mL ± 825 pg/mL—*P* = 0.141, Figure 4(c)).

### 3.6. Combination Does Not Modify Tumour Proliferation or Apoptosis

**Figure 5:**
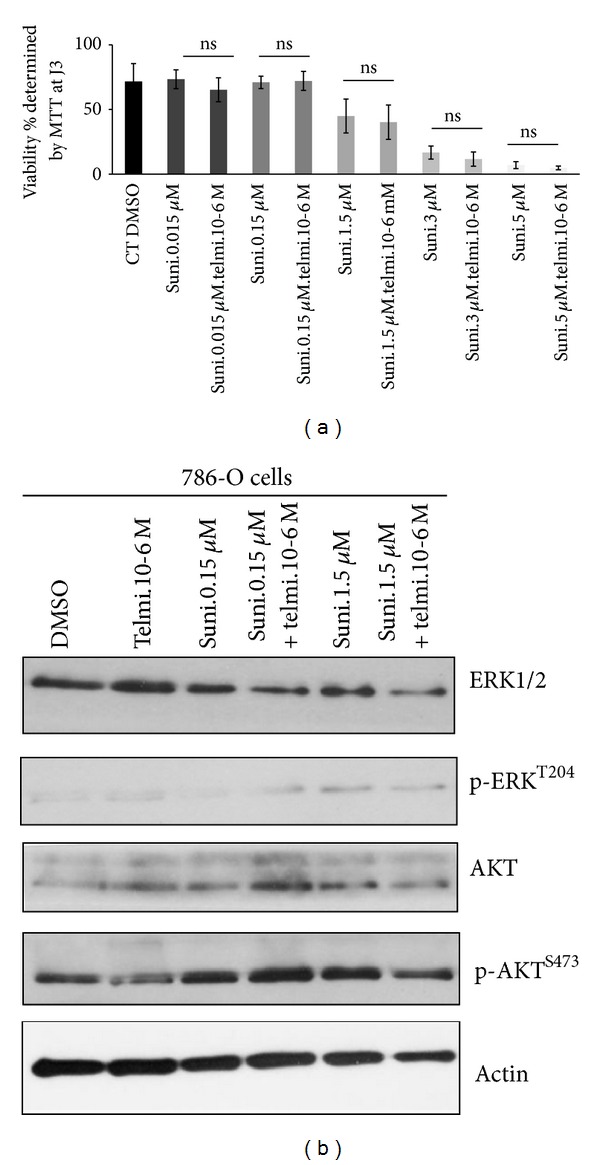
Sunitinib alone or in combination does not modify the expression profile of survival and proliferation pathways. (a) Evaluation of sunitinib toxicity alone or combined with telmisartan by MTT in 786-O cell culture. (b) Western Blot of ERK pathway and AKT pathway in 786-O cell culture.

The effect of combination of telmisartan and sunitinib was first tested on 786-0 cell culture by MTT assay. The association did not modify the effect of sunitinib alone on cell proliferation (Figure 5(a)). Moreover, no variation of the ratio ERK1/2/P-ERK or AKT/p-AKT was registered in the assay (Figure 5(b)). The results indicate that association was not more toxic on 786-O cells than sunitinib alone.

**Figure 6:**
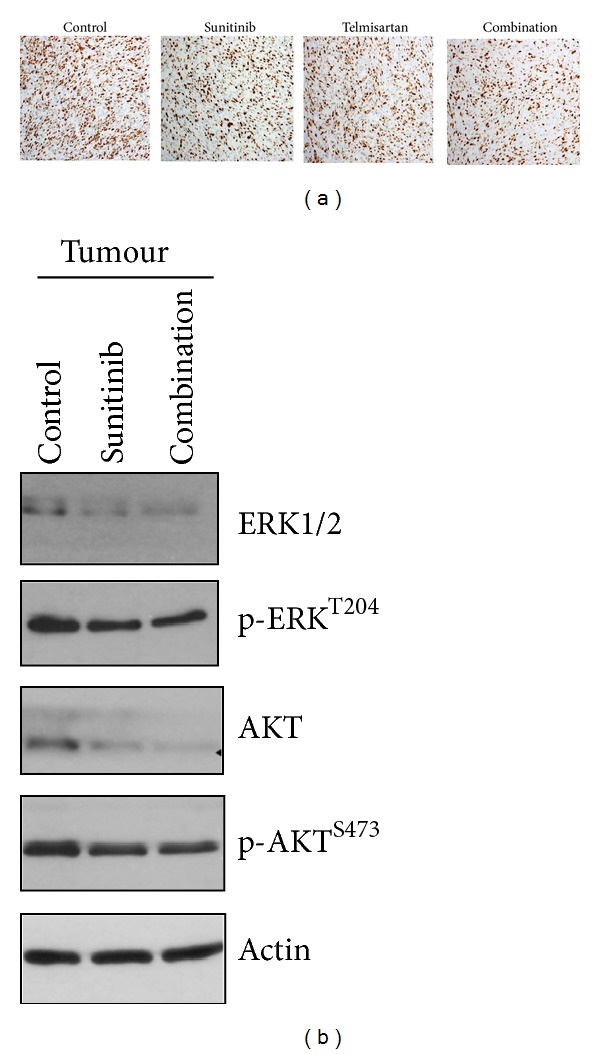
The use of sunitinib and/or telmisartan does not change tumor proliferation or phosphorylation of ERK and AKT. (a) Tumor analysis by Ki67 staining (×200) in control tumor and after treatment with sunitinib alone, telmisartan alone, and in combination. No difference between groups is observed. (b) Analysis of proteins ERK1/2, AkT, p-ERK1/2, and p-AKT from tumor control and treated group.

Telmisartan as well as sunitinib is known to inhibit tumor cell proliferation and induce apoptosis on several tumours. The combination of sunitinib and telmisartan did not inhibit tumor cell proliferation, as well as each of them separately. The percentage of proliferative cells stained by KI67 was analyzed for each group and exhibited a high degree of expression: from 59% to 64% (*P* = ns—Figure 6(a)). The expression of proteins involved in the proliferation and survival pathways was also analyzed by Western Blot (Figure 6(b)). Neither activated ERK pathway nor activated AKT pathway was modified in the case of treatment with the combination of telmisartan and sunitinib.

## 4. Discussion

In this work, we have shown that telmisartan potentiates the effects of sunitinib on renal cell carcinoma by increasing tumor necrosis. AT1-R blockers are widely used drugs. Several years ago, there have been many clinical trials on cardiovascular and renal endpoints, and no increased incidence of cancer has been usually reported as secondary endpoint or in post hoc analysis [15]. Despite those trials and many scientific studies on different types of cancers, there is still a controversy regarding antitumoral or protumoral effects of these treatments [16].

Similar combinations of RAS blockers with classical anticancer therapies have already been studied in other murine models [17–20]. These previous works highlighted the antiangiogenic properties or antiproliferative effects of the associated compounds. Sunitinib was developed to target angiogenesis [21] and AT1-R blockers are well known to decrease angiogenesis in numerous experimental studies [22, 23]. Because of the same therapeutical target, this combination could be seen as redundant but might be beneficial for several reasons. The experimental and clinical studies clearly showed that sunitinib mainly acts by inhibiting VEGFR2 activation on endothelial cells [21] which triggers a negative feedback in tumours leading to a VEGF-A oversecretion [24]. Moreover, AT1-R blockers have been described to inhibit VEGF-A secretion by tumour cells [25] and decrease tumor angiogenesis [22, 26] as well as tumour cell proliferation [27]. AT1-R antagonists could also trigger cancer cell apoptosis [28]. RAS blockers have been studied in many types of cancers, principally prostate, breast, and pancreas, but rarely in ccRCC. Though renal tumors are highly vascularised and could represent one novel target for RAS blockers, only one murine model described that ACE-Is prevent the development of distant metastasis [14].

Some authors clearly showed synergistic association between classical antitumour treatments and AT1-R antagonists or ACE-Is. For example, ACE-Is use increases effects of radiotherapy for the treatment of lung cancer [17]. Similar benefits are obtained in hepatocarcinoma by the association of ACE-Is and 5-fluorouracil [18] or interferon-*β* [19]. AT1-R blockers also improve the efficiency of gemcitabine on pancreatic tumour growth [20]. Based on these data, class effect of antiangiogenesis of angiotensin receptor blockers remains possible.

This multiple step blockage of VEGF pathway leads to the decrease of central neovascularisation and to the increased necrosis observed in our model. Nevertheless, our model does not show any additive effects on proliferation or apoptosis contrary to results of experimental works in other tumor models [28]. It can be suggested that tumors induced by 786-0 cell injection developed molecular mechanisms which confer a resistance against antiproliferative drug regimen. These findings might not be the same in other ccRCC xenograft models and have to be further explored with primary cell lines.

The clinical use of the combination of telmisartan and sunitinib is yet to be determined. In a previous study, we demonstrated that renal cell carcinomas differently express AT1-R with a high expression level of AT1-R in the most aggressive tumors [11]. As described in breast cancer for estrogen receptors [29], it can be expected that tumors that highly express AT1-R will be more sensitive to telmisartan. This hypothesis needs to be confirmed. In addition, some clinical retrospective studies have been performed on patients with cancer receiving RAS blockers for hypertension. Among 127 patients presenting metastatic renal cell carcinoma and treated with sunitinib, the use of ACE-I or AT1-R blockers significantly improved response to treatment and overall survival [30]. The survival of patients with a non-small-cell lung cancer also improves when they receive ACE-I or AT1-R blockers to treat hypertension [31]. The risk of recurrence was also reduced in breast cancer by the use of AT1-R blockers [32]. Prospective study with RAS blockers in addition to anti-VEGF therapies is now warranted.

## 5. Conclusion

Our study demonstrated that telmisartan potentiated antiangiogenic effects of sunitinib in a murine model of xenograft. Combination decreased neovascularisation in the center of the tumor and induced more tumor necrosis. These could be the result of a multiple targeting of different pathways on endothelial cells as well as on tumor cells. Such results must be now confirmed in clinical prospective studies.

## Supplementary Material

The supplementary file describes precisely the method used to develop the kinetic model and its accuracy.
