# Supplementary material for: Sunitinib Combined with Angiotensin-2 Type-1 Receptor Antagonists Induces More Necrosis: A Murine Xenograft Model of Renal Cell Carcinoma
Source: Biomed Res Int. 2014 May 22;2014:901371. doi: 10.1155/2014/901371 (PMC4054801; doi:10.1155/2014/901371)
Supplement: Supplementary file 1 — The supplementary file describes precisely the method used to develop the kinetic model and its accuracy. [file 901371.f1.pdf]

Supplementary data: statistical analysis

All individual data were analyzed simultaneously using nonlinear mixed effect models, which allow sharing information across subjects. A three parameter sigmoidal mixed-effects model was used to describe tumor growth. Parameters were estimated by maximum likelihood (MLE), using the extended stochastic approximation expectation-maximization (SAEM) algorithm as implemented in the saemix package [ref] in the R statistical software (version 2.13, http://www.Rproject.org). Treatments effects on each parameter were tested through three covariates: Telmisartan (ARA2), Sunitinib (TKI) and an interaction term (covariate with value of 1 when ARA2 and TKI are given together, 0 otherwise). A backward model selection procedure with Bayesian Information Criteria (BIC) was used to build the final model. The following diagnostic plots were used to assess visually the model fit (not shown): observed vs. population predicted value, individual predicted profiles superimposed on observations and Visual Predictive Check (VPC), using 1000 simulated datasets. The population parameter estimation for each of four groups with or without ARA2 and TKI are reported for this final model. Empirical Bayes estimates were derived from the population parameters and the observed individual data to predict the progression of each individual tumor.

Ref : *E. Comets, A. Lavenu, M. Lavielle. 20th PAGE congress, June 2011, Athens*

Supplementary data:

Non-linear mixed effect models were used to analyze the longitudinal data. Assuming yij denotes the jth tumor size measured at time x_{ij} in the ith subject (i=1,...N, j=1,...,ni), the statistical model describes the observed tumor sizes through a model f and individual parameters ψ_i according to the following equation:

$$y_{ij} = f(\psi_i, t_{ij}) + g(t_{ij}, \psi_i, \zeta)\varepsilon_{ij}, 1 \le i \le N, 1 \le j \le n_i$$

where the vector of individual parameters in subject i, $\psi_i = (\psi_{i,l}; 1 \le l \le n_\psi) \in R^{n_\psi}$, is a n_ψ-vector which can be written as ψ_i = h (μ; c_i; η_i), a function of μ, an unknown vector of fixed effects, η_i an unknown vector of random effects and $c_i = (c_{im}$; 1 ≤m ≤M), a known vector of M covariates. η_i is assumed to follow a multivariate normal distribution with variance-covariance matrix Ω:

η_i ~ i.i.d. N (0,Ω)

Here h was chosen to be the exponential function so that the ψ_i are log-normally distributed. Assuming for instance a treatment effect of ARA2 on the pth parameter ψ_i^p leads to the following covariate model:

$$\psi_i^p = \begin{cases} \mu_{\psi^p} \exp(\eta_i), when\, not\, given\, ARA2 \\ \mu_{\psi^p} \exp(\beta_{ARA2,\psi^p}) \exp(\eta_i), when\, given\, ARA2 \end{cases}$$

The erros εij are random samples from the standard Gaussian distribution, and the residual error model is defined by a function *g* a possibly depending on additional parameters ξ. A combined residual error model (g=a+bf) has been chosen for g.

In the present analysis, the structural model f was chosen to be the sigmoidal Hill model [ref]:

$$f(\psi_i, t_{ij}) = \text{Size\_max}_i (t_{ij})^{gamma} / \left((\text{T50}_i)^{gamma} + (t_{ij})^{gamma} \right)$$

where ψ_i is the vector of three parameters Size_max, T50 and gamma for subject i.

Ref: *Goutelle S, Maurin M, Rougier F, Barbaut X, Bourguignon L, Ducher M, Maire P*. The Hill equation: a review of its capabilities in pharmacological modelling. Fundam Clin Pharmacol. 2008 Dec;22(6):633-48.

---------------- Fixed effects -----------------

Parameter	Estimate	SE	CV(%)	p-value
Size_max	2245.73	210.00	9.42	-
beta_interaction(Size_max)	-0.15	0.10	67.32	0.07
T50	8.05	0.27	3.39	-
beta_ARA2(T50)	0.09	0.03	36.60	0.00
gamma	5.32	0.42	7.85	-
beta_ARA2(gamma)	-0.24	0.10	43.57	0.01
a	9.99	1.90	18.75	-
b	0.27	0.00	0.01	-

---------- Variance of random effects ----------

Parameter	Estimate	SE	CV(%)
Size_max	0.03	0.01	39
gamma	0.08	0.02	26

Table 1: Estimates of the population parameters and their standard error (SE) and variability (expressed as %) with significant degree for covariate effects on the parameter. The final covariate models are:

$$\text{Size\_max}_i = \text{Size\_max} \times \exp(\text{beta\_interaction(Size\_max)} \times c_{\text{interaction}}) \times \exp(\eta_i),$$

$$\text{T50}_i = \text{T50} \times \exp(\text{beta\_ARA2(T50)} \times c_{\text{ARA2}}) \times \exp(\eta_i),$$

$$gamma_i = gamma \times \exp(\text{beta\_ARA2(gamma)} \times c_{\text{ARA2}}) \times \exp(\eta_i),$$

where $c_{interaction}$ is 1 if the subject receives both ARA2 and TKI together, 0 otherwise,
and c_{ARA2} is 1 if the subject receives ARA2, 0 otherwise

The tumor growth in each mouse was modeled via population analysis (Figure 1bis). The final model includes a positive effect of Telmisartan (ARA2) on the inflexion point (p= 0.0031), a negative effect on the curvature (p=0.0109) and a non significant negative effect (p=0.0687) of Sunitinib (TKI) in addition to Telmisartan (ARA2) on the final size of the tumor (table 1, figure 2). The model suggests that tumor growth is slower with Telmisartan. The trend to a smaller maximal size when both drugs are co-administered can be seen in figure 3 where the group with both drugs has a predicted response lower than that of the other groups over the whole dose range. The predicted size of tumors at 10 and 22 weeks respectively is 1206 and 1864 cc when both drugs are given, 1406 and 2174 cc when Telmisartan is administered alone, and 1707 and 2208 cc respectively in the control group.

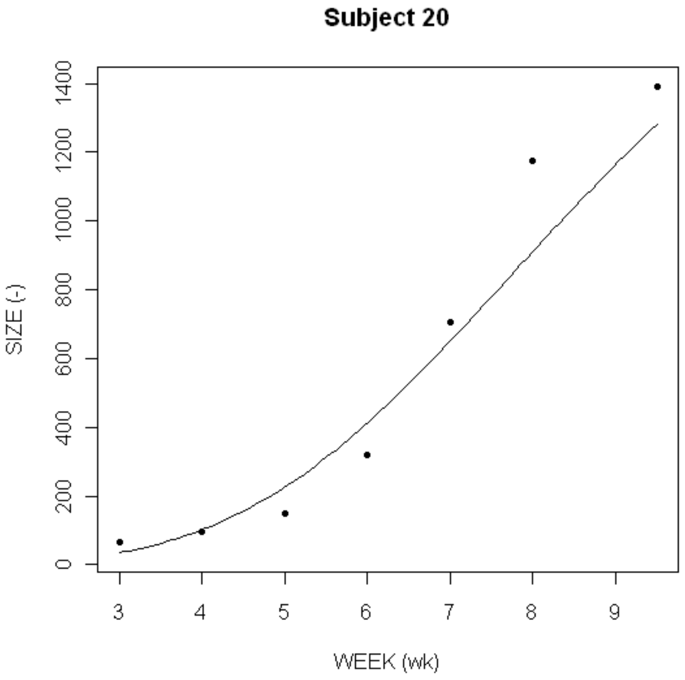

Figure 1 bis: Individual fit of tumor growth for one of the 40 mice.

Group	Size_Max (cc)	T50	gamma
TKI and ARA2	1926°	8.84	4.18
ARA2 alone	2245	8.84*	4.18*
2 groups : TKI alone, and the two drugs	2245	8.05	5.32

*The T50 parameter increases with ARA2 (p= 0.0031) and the gamma parameter decreases with ARA2 (p=0.0109)

° The Size_Max parameter tends (but no significatively) to decrease when TKI is given in addition to ARA2 (p=0.0687)

Table 1: Kinetic parameters of the four groups with or without Telmisartan (ARA2) and Sunitinib (TKI).
